# Youth-Physical Activity Towards Health: evidence and background to the development of the Y-PATH physical activity intervention for adolescents

**DOI:** 10.1186/1471-2458-14-122

**Published:** 2014-02-05

**Authors:** Sarahjane Belton, Wesley O’ Brien, Sarah Meegan, Catherine Woods, Johann Issartel

**Affiliations:** 1 School of Health and Human Performance, Dublin City University, Dublin 9, Ireland; 2 College of Arts, Celtic Studies and Social Sciences, University College Cork, 2 Lucan Place, Western Road, Cork, Ireland

**Keywords:** Physical activity, Adolescents, Intervention, Fundamental movement skills

## Abstract

**Background:**

Despite known benefits of regular physical activity for health and well-being, many studies suggest that levels of physical activity in young people are low, and decline dramatically during adolescence. The purpose of the current research was to gather data on adolescent youth in order to inform the development of a targeted physical activity intervention.

**Methods:**

Cross-sectional data on physical activity levels (using self report and accelerometry), psychological correlates of physical activity, anthropometic characteristics, and the fundamental movement skill proficiency of 256 youth (53% male, 12.40 ± 0.51 years) were collected. A subsample (n = 59) participated in focus group interviews to explore their perceptions of health and identify barriers and motivators to participation in physical activity.

**Results:**

Findings indicate that the majority of youth (67%) were not accumulating the minimum 60 minutes of physical activity recommended daily for health, and that 99.5% did not achieve the fundamental movement skill proficiency expected for their age. Body mass index data showed that 25% of youth were classified as overweight or obese. Self-efficacy and physical activity attitude scores were significantly different (p < 0.05) between low, moderate and high active participants. Active and inactive youth reported differences in their perceived understanding of health and their barriers to physical activity participation, with active youth relating nutrition, exercise, energy and sports with the definition of ‘being healthy’, and inactive youth attributing primarily nutritional concepts to ‘being healthy’.

**Conclusions:**

Data show a need for targeting low levels of physical activity in youth through addressing poor health related activity knowledge and low fundamental movement skill proficiency. The Y-PATH intervention was developed in accordance with the present study findings; details of the intervention format are presented.

## Background

Physical activity (PA) as a preventive measure is widely recognised as central to effective management of overweight and obesity problems [1], and for children it is seen as an important factor in reducing the risk of chronic disease in adulthood [2,3]. Evidence suggests that habitual physical activity (PA) amongst young people is declining with a rise in incidence of overweight and obesity [1,2]. Consequently, children and adolescents are a target population for the promotion of PA to enhance health. A critical consideration for children, adolescents, parents, professionals, and scientists is the implementation and adherence to PA guidelines for health [4]. The most widely endorsed PA guideline stipulates that in order to enhance health, youth should accumulate at least 60 minutes of moderate-to-vigorous PA (≥ 60 min. MVPA) daily [5-7].

Despite the known importance and associated benefits of regular PA in promoting lifelong health and well being, studies suggest that levels of PA decline dramatically during adolescence [8,9], with males significantly more active than females [10-13]. Evidence now emphasises the need for research to generate sound knowledge on models of successful intervention in PA [4]. In their policy guidelines aligned to the Health Behviour in School-aged Children 2012 results, the World Health Organisation (WHO) [13] supported the need for policy interventions to increase PA. The WHO [13] state that policy-makers and practitioners should seek to identify what prevents and what motivates participation. Some of the factors listed as ensuring equitable access in this report include *“providing a range of activities that appeal specifically to girls, ensuring activities are free or affordable, with provision of free or low-cost transportation to the venue, and involving young people in programme design to identify barriers to participation.”* (pp 137) [13].

The Centres for Disease Control and Prevention’s (CDC) Task Force on Community Preventive Services [14] in a systematic review of community interventions designed to increase PA, recommended six different types of intervention as having good evidence for achieving sustainable behaviour change in PA. Consistent with recent findings [15,16], the organisation level or school-based physical education (PE) was highly recommended as one of these intervention types [14]. PE has the opportunity to reach nearly all school-aged children [17], and has been associated with improved mental health, dietary choices and academic achievement [18]. For an increasing number of children PE may be the only opportunity they have during the week to engage in MVPA [19], and as subject area PE is now widely accepted as a public health resource [15]. Increasingly, studies are reporting the positive effect school-based PE interventions have on PA participation [16,20,21].

A systematic review [22] of 76 interventions worldwide aimed at promoting PA participation in children and adolescents found that for children (defined as 4 – 12 years), school-based interventions with a focus on PE and involving school break times were the most effective. For youth (defined as 13 – 17 years old), tailored advice sessions were found to be more effective. A previous systematic evaluation of evidence [23] found that at approximately 10 years of age PA priorities start to change from general PA with an emphasis on motor skill development to prescriptive PA with an emphasis on health, fitness and behavioural outcomes. To this end another review on the effectiveness of PA interventions [24] shows that a large number of PA intervention programmes have reported some element of health education (related to PA) as part of the intervention structure, however, it not clear how young people’s knowledge of (or beliefs and attitudes towards) the role of PA in ensuring optimal health, affects their PA participation. This review [24] reported strong evidence showing that school-based interventions with a family or community component can increase PA in adolescents (defined as ≥ 10 years).

The strategic advice outlined in the Children’s Sports Participation and Physical Activity study [12] in order to achieve the overall recommendation of increasing PA levels of Irish youth include the development and promotion of fundamental movement skills (FMS). Cross-sectional evidence has grown regarding the importance of fundamental movement skill proficiency, showing that it is positively associated with total PA [25], moderate-to-vigorous PA [26], skill-specific PA [27] and organised PA [28] in youth. Mastery of motor skills in childhood is likely to be a key determinant of later adolescent PA [29]. Findings of a recent study [30] provide support for the simultaneous targeting of increasing PA and fundamental movement skills in PA interventions; such is the evidence of a school-based programme [31] which found positive effects in FMS and PA levels following a 5 month respective PE intervention. The CSPPA study [12] found that ‘lack of competence’ was the most common reason cited for non-participation in sport and PA by children and youth. The link between poor FMS levels and low levels of PA have been described above, however what is less apparent in an Irish context is the current levels of FMS of youth, particularly at the critical period of transition from primary to post primary education. Children have the developmental potential to master most of the FMS by 6 years of age [32], yet recent evidence outside of Ireland suggests adolescent youth are not performing FMS to their expected developmental capability [33-35]. This emerging evidence indicates that children are likely making the transition to adolescence without acquiring basic movement skill proficiency, though this has yet to be confirmed in an Irish context.

In order to counteract the attraction of sedentary pursuits, and to promote lifelong engagement in PA, intervention programs need to be developed focusing on the unique needs of young people [4]. To design personally meaningful and socially relevant PA interventions for youth, their views and opinions and insight into motivations and barriers they experience in relation to participation both within and beyond school must be sought. This improved understanding of the factors that influence young people’s physical activity behaviors will allow for the planning of more appropriate interventions to promote PA within this cohort [36]. To simplify this process the Youth Physical Activity Promotion (YPAP) Model [37] guided the development of the Y-PATH intervention. The YPAP model adopts a social-ecological framework acknowledging the input of various personal, social and environmental influences on physical activity. The social-ecological framework acknowledges that self-regulation is difficult to establish without broader social and institutional support [38]. The YPAP model was developed based on aspects of the Precede-Proceed health promotion model [39], which advocates a ‘bottom-up’ approach to program planning, considering the specific characteristics and needs of a population prior to establishing any programme.

**Figure 1:**
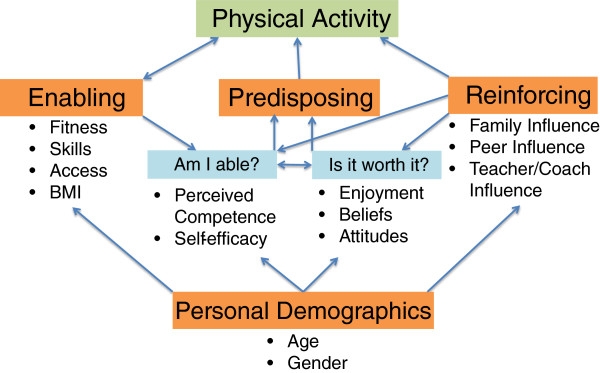
Interrelationships within the Youth Physical Activity Promotion model.

The YPAP model has four major components; enabling factors, predisposing factors, reinforcing factors, and personal demographics. Predisposing factors are identified as those which impact on the decision making processes of youth as they decide whether to engage in, or avoid PA opportunities. These factors include youth’s attitudes towards PA, and their perceptions of the benefits, the level of enjoyment and their competence levels for the particular type of PA offered. The reinforcing factors are those influences that encourage participation through the social environment. Specifically, for youth significant others include parents, teachers/coaches and peers. This network of significant others can provide positive support (e.g. providing transport) and encouragement (e.g. asking about sport engagement) for regular engagement in PA, and in doing so enhance the likelihood of youth engagement in PA opportunites [37]. Enabling factors or personal attributes (such as fitness, FMS, body composition) are recognized in the model as necessary but not sufficient determinants of physical activity. Demographic factors (such as age, gender) are identified as those directly influencing how an individual will assimilate various influences. The YPAP model as applied in the Y-PATH intervention development is presented in Figure 1.

Martínez-Andrés et al. [40] advocate for the use of mixed methods in studies aiming to develop effective interventions for youth, using qualitative methods to better understand young peoples points of view in relation to barriers and motivators for PA, and using quantitative data to get a more objective picture of the amount of PA youth participate in. The purpose of the current study (Y-PATH: Youth Physical Activity Towards Health) was to collect such data in an Irish context so that a meaningful and relevant intervention could be developed. In line with the factors outlined in the YPAP model data on current levels of PA and FMS of 12 – 14 year old adolescent youth were collected, along with data on psychological correlates of PA (including attitude and self-efficacy). Focus group interviews were then used to explore barriers and motivators to PA of the cohort. Based on the information gathered an intervention program ‘Y-PATH’ specifically tailored for the needs of this age group was developed.

## Methods

### Participants and recruitment

As part of a cross-sectional research design all second level schools in a rural Irish town (two mixed schools, one all male school, and one all female school) were targeted and provided consent. All first year students (aged 12 – 14 years) within these four schools were invited to be involved in the study; 256 participants (from a possible total of 288) agreed to participate. Informed consent for participation was granted by each of the 256 participants and their parent/guardian; all participants were free to withdraw from the research at any stage. Full approval for this study was given by the Dublin City University Research Ethics Committee (DCUREC/2010/081).

### Procedures and data management

Body mass (kg) and height (m) were directly measured using a SECA Leicester Portable Height Measure and SECA heavy duty scales. Level of PA participation, and psychological correlates of PA were assessed via self-report (detail given below). A sub sample of participants (one class group from each school, total n = 117) also wore an Actigraph GT1M or GT3X accelerometer for a period of 9 days in order to provide an objective measure of habitual PA participation; due to a firmware malfunction data from almost all of the sub sample failed to download and as such accelerometer data has been omitted analysis. Each FMS was assessed in conjunction with the behavioral components from three established instruments: Test of Gross Motor Development [41], Test of Gross Motor Development 2 [42] and the Victorian Fundamental Motor Skills manual [43]. Focus group (FG) interviews were used to explore students’ perceptions of what it means to be healthy and to identify their motivators and barriers to PA participation (further information given below).

The questionnaire developed for the Y-PATH study was a combination of well-known, valid, and reliable self-report measures. The measures used were developmentally suitable for adolescents of this age group and addressed the key areas of research interest. Habitual PA was assessed using two questions [44] - the number of days during the past week, and for a typical week, that participants accumulated 60 minutes or more of MVPA. A composite average of the 2 items provided a score of days per week that the adolescents had accumulated 60 minutes of MVPA, this method has recently shown moderate to large correlations with objective accelerometer data (0.20-0.51) amongst adolescents [45]. Questions on psychological correlates of PA (self-efficacy, perceived benefits and barriers to participation, PA attitudes, and social support) were all taken, with scales intact, from the FifeActive survey [46].

Data was collected on participants in their class groups (maximum n = 30) during a 2-hour school visit, with a ratio of 1 researcher to 10 students for questionnaire completion, and 1 researcher to 5 students for all other measures. The study was briefly explained and instructions provided on how to complete the questionnaire. Participants were encouraged to take their time, reflect on their answers, and to be as honest as possible. All questionnaires were completed online through ‘Survey Monkey’ in class, with an ID number assigned. In cases where computer networks failed, participants completed hardcopies of the questionnaire. A 48 hour time sampling test-rest reliability measurement among a sample of 35 participants (11–12 years of age) was carried out to ensure comparability of the two administration protocols (computer versus hardcopy); reliability coefficients ranged from 0.81 to 0.94, showing the scores across both formats of the questionnaires to be very stable over time,

FMS data were collected during physical education (PE) classes; again, participants were assigned ID numbers for anonymity purposes. Prior to FMS data analysis, researchers were required to reach a minimum of 95% inter-observer agreement for all 9 skills on pre-coded data. The following 9 FMS were assessed (along with height and weight) during a timetabled 80 minute PE lesson: run, skip, horizontal jump and vertical jump (locomotor; maximum score = 34); kick, catch, overhand throw, strike and stationary dribble (object control; maximum score = 40). Prior to FMS data analysis, researchers were required to reach a minimum of 95% inter-observer agreement for all 9 skills on pre-coded data. FMS analysis focused exclusively on the raw scores across the selected 9 FMS. The number of FMS performance criteria varied from 3 to 6 across the range of selected FMS; all participants were given a ‘1’ for correct execution of criteria, and a ‘0’ for a failed attempt. Participants performed the skill on 3 occasions including 1 familiarization practice and 2 performance trials. For each FMS, the two performance trials were added together to get the total for each skill score. There were a total of 74 performance criteria for all 9 gross motor skills. A Total score for all 9 skills was calculated for each participant, along with an object control score, and a locomotor score.

FG interviews that explored student perceptions of what it means to be healthy and their views on the important factors that influenced their involvement in, or avoidance of, PA were also conducted. A semi-structured interview guide, using questions designed by the research team whom had specific expertise in qualitative design and PA intervention development were developed. Questions were piloted on a sample of 16 students from the target group. Following the pilot work, 8 FG interviews were conducted in the 4 schools post self-report and FMS data collection. Each school had 2 focus group interviews, 1 with a ‘high active’ group and 1 with an ‘inactive’ group. A subsample of students (n = 59) randomly selected from the 4 schools were selected into either ‘high active’ or ‘inactive’ categories based on the previously collected self-report data at baseline (0–3 days 60 minutes MVPA = inactive; 6–7 days 60 minutes MVPA = high active). The rationale for this was, in line with a previous study [47], that students were more likely to contribute to discussions about PA if homogeneously grouped in terms of PA participation levels. Prior to FG commencement, all participants received and signed a consent form and a plain language statement providing details of the research. Focus groups occurred in a school classroom and lasted an average of 45 minutes. Participants were reminded that they could withdraw from the interview at any stage and that all recordings would remain confidential. The FG interviews were recorded by Dictaphone and were transcribed verbatim. Each of the eight FG interviews were conducted by two researchers; a facilitator and a note taker. The facilitator’s role was to guide the FG, stimulate interaction among students toward the theme, oversee group discussion and encourage all students to respond. The note taker kept a record of the discussion as it evolved to add details for instances where the recording was not audible [47].

### Data analysis

All data were analysed using SPSS version 20.0 with alpha set at p < 0.05. Where participants had incomplete data for a given variable, participants were excluded from analysis of this variable specifically. The number of days self-reported meeting the 60 minutes PA guideline were analysed descriptively using means, standard deviations and proportions. Students were categorised as low active (meeting guidelines on 0, 1, 2 or 3 days a week), moderate active (meeting on 4 or 5 days), or high active (meeting on 6 or 7 days). A summative score was calculated for each psychological determinant (scoring system detailed in FifeActive [46]). Descriptive statistics and frequencies for all FMS were calculated. “Mastery” was defined as correct performance of all skill components on both trials, “Near Mastery” was defined as correct performance of all components but one on both trials [48]. As the assumptions for ANOVA were met, two-way between groups ANOVA’s were used to explore the impact of gender and PA grouping (low, moderate or high active) on total FMS score, locomotor score, object control score, BMI, and on all psychological correlates. In instances where significant main effects were found, post-hoc comparisons were be carried out using the Tukey HSD to determine where the differences occurred.

The FG data was analyzed using the constant comparative method [49]. This process involved manually highlighting and comparing the emergent themes from the associated data collected in the FG interviews. Similar themes from the high active and inactive focus group participants were grouped together under several headings. Areas of significance and importance in relation to students’ perception of health and their views on the factors, deemed important in motivating or preventing their participation in PA, were identified. In order to ensure data trustworthiness and credibility, various steps were taken including member checking, peer examination and independent data coding. Member checking involved the researchers discussing the main findings with the FG participants to verify accurate reflections of the discussion [49]. Participants were given the opportunity to make amendments or add suggestions. No participants made any changes to the research findings. Peer examination of the data occurred between the researchers to ensure individual researchers found similar trends from the data and a second reader independently coded the data. Differences in the coding were discussed between the researchers and independent coder until consensus was reached.

## Results

Table 1 gives the gender breakdown of mean (SD), along with the sample size, for all variables, both overall and across PA grouping.

**Table 1:** Mean (SD) across PA grouping with significant main effects and effect size of two-way ANOVA’s

				**Physical activity grouping**				
**Variable**	**Gender**	**N**	**Overall**	**Low**	**Moderate**	**High**	**Main effect**	**Partial eta squared**
BMI	Male	131	19.89 (3.45)	20.60 (4.32)	19.61 (3.04)	19.93 (3.25)	No sig.	
	Female	113	20.17 (3.13)	20.26 (2.77)	20.16 (3.28)	19.79 (3.49)		
Total FMS	Male	120	61.93 (5.56)	60.81 (6.03)	62.51 (6.76)	62.18 (4.14)	No sig.	
	Female	103	60.13 (5.33)	60.73 (5.14)	60.60 (4.90)	59.96 (5.30)		
Object control	Male	110	36.62 (2.62)	36.13 (2.81)	36.49 (3.17)	36.75 (1.97)	***Gender***, F(1,199) = 9.147, *p* = 0.003	0.044
	Female	95	35.27 (2.82)	35.30 (2.93)	35.57 (2.71)	35.04 (2.47)		
Loco-motor	Male	120	25.31 (4.19)	24.68 (4.61)	26.03 (4.63)	25.43 (3.39)	No sig.	
	Female	103	24.85 (3.94)	25.43 (3.45)	25.03 (3.67)	24.93 (4.16)		
Self efficacy	Male	133	25.36 (4.04)	23.74 (41.8)	26.30 (4.17)	25.73 (3.47)	***PA***, F(2,245) = 4.087, *p* = 0.018	0.032
	Female	118	24.80 (4.27)	24.28 (4.58)	24.74 (4.20)	25.56 (3.90)		
Benefits	Male	133	31.71 (4.04)	31.23 (4.80)	32.11 (3.88)	31.71 (3.55)	***PA***, F(2,245) = 3.513, *p* = 0.031	0.028
	Female	118	31.31 (4.24)	30.19 (4.23)	32.68 (3.40)	31.27 (4.23)		
Barriers	Male	133	33.86 (8.12)	32.63 (7.85)	35.07 (7.05)	33.67 (9.21)	***PA***, F(2,245) = 3.650, *p* = 0.027	0.029
	Female	118	33.91 (6.57)	31.76 (6.61)	34.29 (5.51)	36.38 (6.81)		
Attitudes	Male	129	13.78 (2.30)	12.87 (2.43)	14.09 (2.16)	14.18 (2.17)	***PA***, F(2,240) = 11.332, *p* = 0.000	0.086
	Female	118	33.91 (6.57)	31.76 (6.61)	34.29 (5.51)	36.38 (6.81)		
Social support	Male	129	16.14 (3.94)	15.89 (4.03)	15.88 (3.44)	16.55 (4.32)	No Sig.	
	Female	117	15.90 (3.12)	15.52 (2.99)	16.57 (3.32)	15.68 (3.04)		

### Descriptive and anthropometric characteristics

Of the 256 participants involved in this study, 53% were male and 47% were female, with a mean (± sd) age of 12.40 ± 0.51 years. Just over half (52%) of participants were attending a mixed school, with 25% attending an all male school, and 23% attending an all female school. Participants had an average BMI of 20.03 ± 3.30 kg/m^2^, with 75% categorised as normal weight, 21% overweight, and 4% obese using the Cole at al. [50] classification.

### PA (self-report)

Self-report PA data showed that 20% of participants met the 60-minute guideline on 0 – 3 days a week (low active), with 31% meeting the guideline on 4 or 5 days (moderate active); the remaining 49% of participants met the guideline on 6 or 7 days (high active). The percentage of participants meeting the guideline on all 7 days was 33%. Results of the two-way ANOVA’s exploring the impact of gender and PA grouping on the different variables are given in Table 1. The interaction effect for gender and PA grouping was not significant for any of the variables. Statistically significant main effects for gender and/or PA grouping is shown in the main effect column in Table 1, along with the effect size (strength of association - Partial Eta Squared) in each case.

### Psychological correlates (self-report)

Of the psychological variables only social support showed no significant main effect for either gender or PA category. All other psychological variables demonstrated a significant main effect for PA but not for gender, with a small effect size in each case (see Table 1). Post-hoc comparisons using the Tukey HSD showed that in each case the mean score for the low active group was significantly lower than the high active group; only in the Barriers to Self-Efficacy and the Attitudes to PA variables were the low active group mean scores also significantly lower than the moderate active groups.

### FMS

Only one participant (0.5%) possessed complete mastery level across all 9 object related and locomotor movement skills, with 11% scoring mastery or near mastery across the 9 skills (see Table 2). The poorest performances were for the vertical and horizontal jumps (locomotor) where 13% and 29% respectively achieved mastery and 10% and 28% achieved near mastery. Results of a two-Way ANOVA showed that while there was no main effect for PA grouping, male participants obtained a significantly higher object control score compared to female participants (p = 0.003). There was, however, no significant gender (or PA grouping) difference in the overall locomotor mean score performance.

**Table 2:** Percentage and raw score, and 95% confidence intervals (95% CI) of mastery of FMS

	**Mastery**		**Raw score (SD)**	
**Locomotor**	**Male**	**Female**	**Male**	**Female**
Run	95.1% (89.2, 98)	76% (66.2, 83.7)	7.92 (0.40)	7.45 (1.09)
Skip	10.6% (6, 17.7)	11% (6, 19.2)	3.67 (1.36)	4.18 (0.95)
Horizontal jump	35.8% (27.5, 45)	20% (12.9, 29.4)	6.30 (1.64)	5.22 (1.93)
Vertical jump	13.8% (8.5, 21.5)	12% (6.6, 20.4)	7.46 (2.68)	7.94 (2.00)
**Object control**				
Catch	70.7% (61.7, 78.4)	64% (53.7, 73.2)	5.57 (0.82)	5.55 (0.66)
Overhand throw	60.2% (50.9, 68.8)	27% (18.9, 36.7)	7.08 (1.33)	6.00 (1.58)
Stationary dribble	65.9% (56.7, 74)	55% (44.8, 64.9)	7.05 (1.62)	6.87 (1.55)
Strike	43.1% (34.3, 52.3)	55% (44.8, 64.9)	9.05 (0.94)	9.25 (0.99)
Kick	86.2% (78.5, 91.5)	78% (68.4, 85.4)	7.80 (0.52)	7.64 (0.76)

### Focus groups

Three key themes emerged from the FG data that were pertinent to students perception of health and what students deemed important in influencing their participation in, and barriers to, PA, PE, sport and exercise These themes were:(1) Being healthy: diet, exercise and body image, (2) Motivators: PA is fun, and (3) Barriers: lack of time, distance, PE related factors.

#### Being healthy: diet, exercise and body image

High active participants perceived being healthy to be related to eating and exercising. One student commented that being healthy means: *‘eating healthy and you see people jogging on the road and you know they are fit and healthy.’* Similarly, another student linked being healthy with: *‘well say if you are not doing exercise and you eat too much fattening foods, it will clog up your heart and you will get a heart attack.’*

Inactive participants’ perception of health differed as they largely associated being healthy with nutrition and body image, with exercise rarely mentioned. To illustrate this, one participant associated being healthy as *‘not getting fat…eating the right food to help your body’.* When this response was probed, the same participant went on to explain that eating the right food consists of *‘replacing junk foods with fruit and vegetables’.* Other responses from the inactive participants who associated being healthy with body image included: ‘*you don’t have to worry about being fat’* and being healthy *‘kind of keeps you skinny’.*

#### Motivators: PA is fun

The key emergent theme in relation to motivators for PA among high active participants was enjoyment and fun. Examples of this include a student stating: *‘I think it’s [physical activity] fun and like if you’re at home all day, its great to get out for a couple of hours.’* Similarly, another student stated: *‘well I enjoy it and I know it’s very good and like healthy for you’*. Perceiving physical activity as ‘being fun’ was similarly attributed by inactive participants as a participation motivator, with one student stating: ‘*I just find them fun, they’re good to do’.*

#### Barriers: lack of time, distance, PE related factors

In relation to barriers to PA participation, insufficient time to participate was identified as the main barrier amongst the high active group. One student indicated: *‘we don’t really have any time to do extra sport apart from like football training cos you get back from school at like quarter to 5, you get the bus from here to [place name] so you just kind of get time to eat your dinner, get changed for training, go training, and go home and do your homework’.* Findings illustrated that barriers to PA participation amongst the inactive participants outweighed the motivating factors of their high active counterparts. Several inactive participants cited distance to activity as a barrier to PA participation with one participant stating *‘you travel for sometimes an hour, all the way just to play a match, and then you lose, and it’s a waste of time pretty much.’* Other identified barriers to participation were PE related factors and included the apparent competitive nature, and perceived lack of choice, in PE class. One such participant stated: *‘the guys played and they just got really competitive and it was not fair.’* Another student voiced concerns about the choice in PE: *‘It’s very like, only the team can play, you can’t really choose how you want to do it, and you don’t get to choose what you do.’*

## Discussion

Results of this study highlight that a large number of Irish youth are insufficiently active to benefit their current and future health (only 33% meeting PA guideline for health). Though higher than the 19% reported for a slightly older age group in the CSPPA study [12], this finding is relatively consistent with the range of findings reported for other European countries in the HSBC study [13]. Given the consistently reported decline in activity with age [8,9], the need for intervention to address these low levels reported for young people aged 12 – 14 years old is evident.

The majority of youth in this study (99.5%) failed to reach a level of mastery across key FMS, indicating that basic movement skill proficiency is low. Other research outside of Ireland examining the FMS proficiency of adolescents support this low level of FMS mastery [34,51]. Guided by previous findings [22], it is important to recognise that an intervention designed around movement skill acquisition alone would probably be insufficient to change PA behaviour in youth long term. This points to the targeting of an improvement in FMS proficiency as a strategic supplement in the promotion of PA in adolescents.

Consistent with other studies [36,52-56], analysis of psychological variables reveals an association with PA level. This was evident for self-efficacy, perceived barriers and benefits, and attitude towards PA, all of which exhibited moderate effects. At the individual level, the CDC [3] recognises that for young people perceived competence, and perceptions of their ability to perform a PA (self-efficacy), will affect their participation in an activity. This is also consistent with recent reviews [3,52,53,57], where self-efficacy was found to be a consistent positive determinant of PA in children and adolescents. Like self-efficacy, a significant difference was found in the attitude to PA variable between low and moderately active, and low and high active participants; with low active participants scoring significantly lower than their moderate and high active counterparts in each case. These variables (attitude, self-efficacy) are categorised as predisposing factors for PA in the YPAP model [37], indicating they have a strong influence on the likelihood that a young person will become physically active. It is worth noting that social support (addressing the reinforcing factors presented in the YPAP model [37]) was not found to be significantly associated with PA level in this study. However its previously identified importance in intervention design [58,59] and as a correlate of PA behaviour [52-57] cannot be discounted. The evaluation of social support in the pilot study was conducted using the social support items from the FifeActive survey [46], it is very possible that had a stand-alone social support instrument (such as that presented in Sallis et al. [60]) been used in this research a significant association with PA level may have been found.

From the FG findings, it was evident that the high active and inactive participants had different perceptions of health, and the relative contribution of physical activity to that concept. High active participants cited the importance of ‘exercise’, which supports previous research [61] that found that students’ responses similarly linked practices like eating and exercise with being healthy. Findings from the FGs suggest however that inactive participants associate the term health with body images such as being skinny and avoiding becoming fat. Similar research [62] also found that children perceived being fit and healthy as being skinny and losing weight.

As was found in a previous study [63], both high active and inactive participants identified PA being fun as a primary motivator for participation. Participation barriers identified by inactive participants included lack of time [64], distance [65], the competitive nature of PE, and lack of choice in PE. Research examining the environmental influences on PA among adolescents [66] similarly found that competition was one of the predominant barriers to students fully participating in PE. Other research suggests offering alternate, non-competitive forms of PE as realistic ways for change and improving the long-term participation in PE for children and youth [47,67].

Findings of this study clearly highlight the need for intervention to improve PA levels of young people, and provide good insight into how we can best structure the intervention so that it is most meaningful and relevant. Specifically findings point to the need to:

1) Target both low locomotor and object control FMS levels.

2) Build PA opportunities that help children to develop positive predisposing factors for PA engagement, specifically exercise self-efficacy and attitude.

3) Ensure PE class consists of choice and is primarily cooperative rather than competitive

4) Educate on the health benefits of PA

It has been acknowledged widely in the literature that there is strong rationale for school-based programmes aimed at increasing PA levels [4,23,68], FMS levels [31] and reducing inactivity [3]. Schools have direct contact with children and youth for on average 6 hours per day, and for up to 13 years of their critical social, psychological, physical and intellectual development [3]. In the 2012 follow up paper to their 1991 publication describing the importance of PE in addressing public health problems [15], authors reiterate their recommendation of the following two goals for health related PE to (i) prepare youth for a lifetime of PA, and (ii) provide them with opportunities for PA participation during PE classes. Welk [37] states that PE is recognised as an optimal vehicle for influencing PA habits of young people, specifically paying a primary role in influencing the enabling and predisposing factors identified in the YPAP model. Through education in PE class youth have the potential to be influenced to adopt a physically active lifestyle, with much research showing that activity levels in youth track into adulthood [58,69].

Based on the findings of this study it is apparent that a large number of students were insufficiently active and insufficiently skilled to benefit their current and future health. Inactive students did not demonstrate the same depth of knowledge of the health benefits of PA as did the high active students, and they demonstrated significantly lower scores for Self-Efficacy and Attitude than their moderate and high active counterparts. As such the Y-PATH intervention was developed with a strong focus on physical education based Health Related Activity (HRA), with key school, teacher and parent components. The CDC [59] recommend the promotion of PA through coordinated school health programmes such as this, with links established between the school and the family. This is in line with the YPAP model (see Figure 1), and is strongly supported by a recent systematic review of PA interventions for adolescents, which suggests the importance of targeting ecological domains beyond the individual level [70]. It also encompasses lessons learned from the current study, which recommended enhancement of our intervention’s social support element and the use of more detailed questionnaires to evaluate the impact of social support in any future research evaluating the effect of the Y-PATH intervention.

**Figure 2:**
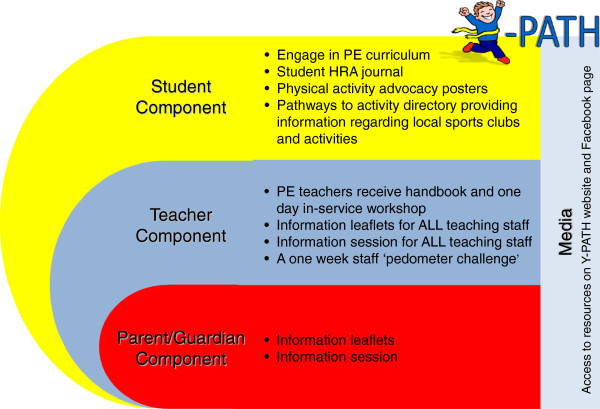
Overview of the structure of the Y-PATH programme.

The Y-PATH intervention was designed in line with the YPAP model with a view to enabling youth to positively re-evaluate their predisposing factors ‘Am I able’ (e.g. self-efficacy) and reinforcing factors ‘Is it worth it’ (e.g. enjoyment, attitudes), while also addressing the enabling factors (e.g. skill level) that influence participation. An overview of the structure of the Y-PATH programme is given in Figure 2. Detail on the student, teacher and parents components of the intervention are given below. The guiding principles of the Y-PATH intervention are then detailed, in each case the relevant component of the YPAP model that is being addressed is identified.

### Student component

A printed Y-PATH resource comprising of two main parts was developed for PE teachers in the intervention school. The first part is a six-lesson scheme of resources. Within each lesson there is both a direct HRA and PA focus, and also a targeted psychosocial focus aimed at improving self-efficacy and attitude towards PA of the students. The second component is a resource to guide PE teachers in the integration of HRA, psychosocial, and FMS components across the remaining seven strands of the Irish Junior Cycle Physical Education curriculum. The importance of teaching strategies within PE classes cannot be overlooked as an important component of the intervention, with Rosenkranz et al. [17] and Ntoumanis [71] indicating that teachers should use motivational strategies within PE classes to support students’ needs, and thus generate a climate where students feel more self-determined to participate in PE. The authors highlight four such motivational strategies: (1) “choice” providing students with the opportunity to make decisions about the activities they participate in during lessons; (2) “relevance” providing a rationale and explaining to students the relevance of an activity; (3) “acknowledgement” acknowledging students’ difficulties when learning skills; and (4) “feedback” providing feedback using praise for students’ effort and improvement.

PE teachers receive a one-day in-service workshop to train them in delivery of the Y-PATH programme with their first year class groups, with specific pedagogical emphasis on the use of motivational strategies within their teaching such as those detailed above. The TARGET model of creating a mastery motivational climate as proposed by Ntoumanis & Biddle [72] was used as a basis for this. The focus of the workshop is to identify with teachers the need to move PE classes from a performance-involving climate (characterised by normative competition, students’ worried about mistakes, and an orientation to succeed with little effort [73]), to a motivational climate (characterized by cooperative learning and a focus on individual improvement, with students oriented towards developing new skills, and improving levels of competence and sense of mastery [74]).

A printed handbook is provided to the students supporting all of the PE teachers’ resources, including a PA log book, which is used within PE class by students to periodically monitor their PA levels. In addition to this teachers receive four posters to display in or around the school sports hall, the focus of these were increasing PA and decreasing sedentary behavior.

### Teacher component

The teacher aspect of the intervention targets all non-specialist PE teachers of the school, challenging all to become facilitators of PA and active role models for young people. Two one-hour workshops are held with all teachers in the school at the beginning and midpoint of the academic school year. During the first workshop, Y-PATH information leaflets are distributed to reinforce the importance of PA and PA promotion and to offer suggestions for how adults can influence youth PA by becoming active role models and encouraging PA. Teachers are then lead by the Y-PATH researcher to develop a charter for the promotion of PA with their students, which is subsequently, given to the school following the workshop. The second teacher workshop explores the ‘voice’ of the teacher in terms of PA promotion.

### Parents component

The parent/guardian aspect of the intervention involves a face-to-face meeting between a member of the Y-PATH research team and the parents of first year students in the school. This is held at the beginning of the academic school year. In this session parents (and students in some instances, depending on school policy) are introduced to the Whole-School Y-PATH intervention as an integral aspect of the school first year programme. Similar to the first teacher workshop parents are directly informed about the importance of PA, information on strategies they can use to promote youth PA, and are given the Y-PATH information leaflets.

### Y-PATH guiding principles

1. The first experience of Physical Education (PE) for the students at second level school will be Health Related Activity, with a focus on PA participation *[move from PE being associated with a specific activity or sport, to being associated with learning to be active; YPAP Predisposing factors].*

2. PE lessons will focus on improving students’ attitude towards PA, self efficacy and fundamental movement skill levels. *[YPAP Enabling and Predisposing factors]*.

3. The climate in PE lessons will be motivational- all students learn that they can be active, experience a range of choice, and learn to challenge themselves and experience success within their own parameters *[focus on attitude and efficacy; YPAP Predisposing factors].*

4. Parents/guardians and non-specialist PE teachers will be targeted as role models that can have a significant influence on students’ attitudes towards PA participation *(move from traditional notion of PE teacher being the person in the school with sole responsibility for health and PA; YPAP Reinforcing factors).*

## Conclusions

Based on the findings of the cross sectional exploratory research presented in this study, a review of the relevant literature, and guided by the YPAP model, the Y-PATH intervention has been developed. It offers a feasible, targeted approach to increasing adolescent PA through a multi-component school based intervention. Future research must examine the efficacy of this evidence-based intervention in a randomized controlled trial, prior to widespread dissemination.

## Competing interests

The authors declare that they have no competing interests, either financial or non-financial.

## Authors’ contributions

SB conceived of the study, participated in its design and coordination, analysed PA data, and drafted the manuscript. WOB participated in study design, coordinated data collection and processing, analysed FMS data, and helped to draft the manuscript. CW participated in study design, coordinated psychosocial data processing and analysis, and reviewed manuscript. SM participated in design of qualitative component of the study, coordinated focus group data analysis, and reviewed manuscript. JI participated in study design and coordination, oversaw FMS data collection, reduction and analysis, and reviewed manuscript. All authors read and approved the final manuscript.

## Pre-publication history

The pre-publication history for this paper can be accessed here:

http://www.biomedcentral.com/1471-2458/14/122/prepub
